# Efficacy of Combined Therapy with Drug-Eluting Beads-Transcatheter Arterial Chemoembolization Followed by Conventional Transcatheter Arterial Chemoembolization for Unresectable Hepatocellular Carcinoma: A Multi-Center Study

**DOI:** 10.3390/cancers13184605

**Published:** 2021-09-14

**Authors:** Asahiro Morishita, Joji Tani, Takako Nomura, Kei Takuma, Mai Nakahara, Kyoko Oura, Tomoko Tadokoro, Koji Fujita, Tingting Shi, Hiroki Yamana, Takanori Matsui, Tadayuki Takata, Takayuki Sanomura, Yoshihiro Nishiyama, Takashi Himoto, Tetsu Tomonari, Akio Moriya, Tomonori Senoo, Koichi Takaguchi, Tsutomu Masaki

**Affiliations:** 1 Department of Gastroenterology and Neurology, Kagawa University, Kita-gun 761-0793, Japan; georget@med.kagawa-u.ac.jp (J.T.); takako-n@med.kagawa-u.ac.jp (T.N.); k-takuma@med.kagawa-u.ac.jp (K.T.); m-nakahara@med.kagawa-u.ac.jp (M.N.); kyoko_oura@med.kagawa-u.ac.jp (K.O.); t-nishioka@med.kagawa-u.ac.jp (T.T.); 92m7v9@med.kagawa-u.ac.jp (K.F.); shitingtingc@med.kagawa-u.ac.jp (T.S.); yamanahiroki@med.kagawa-u.ac.jp (H.Y.); s16d729@stu.kagawa-u.ac.jp (T.M.); takata.tadayuki@kagawa-u.ac.jp (T.T.); tmasaki@med.kagawa-u.ac.jp (T.M.); 2 Department of Radiology, Kagawa University, Kita-gun 761-0793, Japan; sanomura@med.kagawa-u.ac.jp (T.S.); nisiyosi@med.kagawa-u.ac.jp (Y.N.); 3 Department of Medical Technology, Kagawa Prefectural University of Health Sciences, Takamatsu 761-0123, Japan; himoto@chs.pref.kagawa.jp; 4 Department of Gastroenterology and Oncology, Institute of Biomedical Sciences, Graduate School of Medicine, Tokushima University, Tokushima 770-8503, Japan; tetsu.tomonari@gmail.com; 5 Department of Gastroenterology, Mitoyo General Hospital, Kanonji 769-1695, Japan; amoriya@mitoyo-hosp.jp; 6 Department of Hepatology, Kagawa Prefectural Central Hospital, Takamatsu 760-8557, Japan; t-senoo@chp-kagawa.jp (T.S.); k.takaguchi@chp-kagawa.jp (K.T.)

**Keywords:** DEB-TACE, cTACE, Barcelona Clinic Liver Cancer staging system, combination therapy, HCC

## Abstract

**Simple Summary:**

Drug-eluting beads-transcatheter chemoembolization (DEB-TACE) has recently been performed. However, local recurrence of HCC at the tumor margins is often observed. Conventional transcatheter chemoembolization (cTACE) comprises accumulating lipiodol-containing anticancer drugs into the drainage area, which is the first invasive site of hepatocellular carcinoma (HCC). We evaluate the therapeutic effect of DEB-TACE followed by cTACE in patients with intermediate stage HCC. HCC patients were divided into two groups: one group received DEB-TACE followed by cTACE (cTACE group) and the other group received only DEB-TACE (non-cTACE group). The complete response (CR) rate was significantly higher in the cTACE group than in the non-TACE group. The only factor that increased the complete response rate in the cTACE group was the number of tumors. The overall survival (OS) rate of CR patients was higher than that of non-CR patients in the cTACE group. cTACE group adverse events included severe thrombocytopenia but only in one patient. The combined therapy with DEB-TACE followed by cTACE may be useful for HCC patients.

**Abstract:**

EB-TACE has recently been performed because of its lower hepatotoxicity compared to cTACE in less advanced HCC. However, local recurrence at the tumor margins is often observed after DEB-TACE. cTACE involves filling the intratumoral sinusoids with lipiodol-containing anticancer drugs and accumulating in the drainage area, which is the first site of HCC recurrence. The aim of this study is to evaluate the therapeutic effect of DEB-TACE followed by cTACE in HCC patients. Between 2014 and 2020, 65 patients with Barcelona clinic liver cancer (BCLC) stage B (intermediate stage) of HCC were enrolled and divided into two groups: one group received DEB-TACE followed by cTACE (cTACE group) and the other group received only DEB-TACE (non-cTACE group). Sixty-five patients were medically followed. The median observation time was 14 ± 13.1 months after the first DEB-TACE and outcomes were analyzed for multiple factors. Results: The complete response rate was significantly higher in the cTACE group than in the non-TACE group. The analysis showed that the only factor that increased the CR rate in the cTACE group was the total tumor number (less than four). The OS rate of CR patients was higher than that of non-CR patients in the cTACE group. Adverse events in the cTACE group included severe thrombocytopenia but only in one of twenty-seven patients. Conclusions: The combined therapy with DEB-TACE followed by cTACE may be a new effective therapeutic strategy for the intermediate stage of HCC patients.

## 1. Introduction

HCC is the most common cancer [1,2] and the fourth-leading cause of death worldwide [3,4,5,6]. Patient prognosis remains poor due to a lack of effective therapy [7]. While surgery is the most effective therapy for HCC [8], most patients are diagnosed at advanced stages precluding surgical therapy. For these patients, the conventional therapies are cTACE [9], radiofrequency ablation [10], molecular-targeting drugs, or these in combination therapies [2,11].

The Barcelona Clinic Liver Cancer Staging System [12] is widely used to evaluate the staging and consequential treatment of HCC. To treat the intermediate stage of HCC, cTACE is often performed [2,13]. Recently, drug-eluting beads-transcatheter chemoembolization (DEB-TACE) has been recognized as an alternative therapy for patients with advanced, unresectable liver cancer [14,15]. During DEB-TACE, the patient is injected with chemotherapy-loaded microbeads which embolize the arteries that feed the tumor; the drug-loaded beads slowly release a chemotherapeutic agent into the tumor with a systemic drug concentration peak of less than that in cTACE [16]. DEB-TACE reduces the risk of drug-related adverse events such as post-embolization syndrome [1,17]. Zhiyi et al. demonstrated that DEB-TACE treatment achieved a 19.9% complete response (CR) rate and a 79.6% objective response rate [18]. However, after DEB-TACE, residual areas are often found at the tumor margins, at which increased local recurrence can cause serious damage to the patient. In other words, this residual area, at which drug-eluting beads cannot reach, reduces the CR rate. Therefore, cTACE [19], which is effective for this residual region, is performed as a second line treatment. The aim of our study was to evaluate the efficacy and safety of combination therapy with DEB-TACE followed by cTACE in a short term for the treatment of the intermediate stage of unresectable liver cancer.

## 2. Methods

### 2.1. Patient Selection and Eligibility

This multiple-institution retrospective study was approved by the Institutional Ethics Committee of the Kagawa University, Faculty of Medicine (Kita-gun, Japan), in accordance with the Declaration of Helsinki (approval number 2019-271, approved on March 31, 2020). Sixty-five patients with HCC who underwent DEB-TACE between 2014 and 2020 at the Kagawa University Hospital (Kita-gun, Japan), Tokushima University Hospital (Tokushima, Japan), Mitoyo General Hospital (Mitoyo-shi, Japan), and Kagawa Prefectural Central Hospital (Takamatsu, Japan) were examined for this retrospective study. The requirement for informed consent from the participants was waived because of the retrospective nature of the study. The study inclusion criteria were: (i) patients diagnosed with the intermediate stage of hepatocellular carcinoma in accordance with the Barcelona Clinic Liver Cancer Staging System [2]; (ii) patients over 18 years old; and (iii) patients who had received DEB-TACE treatment. The exclusion criteria were: (i) patients with advanced stage liver cancer; (ii) patients who were lost to follow-up; (iii) patients with liver or renal failure; and (iv) patients with an allergy to chemoembolization reagents. The patient data collected in our study also included sex, age, etiology of cirrhosis, the Child-Pugh score, clinical tumor stage (cStage), macroscopic classification, up-to-7 criteria, alpha fetoprotein (AFP), the third electrophoretic form of lentil lectin-reactive AFP (AFP-L3) [20], and des-gamma-carboxy prothrombin (DCP) [21] (Table 1).

### 2.2. DEB-TACE Procedures

After the supernatant was extracted from one bottle of drug-eluting beads (DC beads; Eisai Co. Ltd., Tokyo, Japan), one vial (2 mL) of drug-eluting beads with diameters of 100–300 μm was loaded with 50 mg of epirubicin (Nippon Kayaku Co. Ltd., Tokyo, Japan) and diluted 10 times with iopamidol 300 mgI/mL (Iopamiron 300; Bayer Schering Pharma, Osaka, Japan) [22]. Each drug-eluting bead was transferred to a 20-mL syringe in the significant diffusion state and injected at 1 mL/min. A microcatheter that was 130 cm in length (Progreat^®^; Terumo Co. Ltd., Tokyo, Japan) was used. The outer diameters of the catheter tip and shaft were 1.7 and 2.8 Fr, and the inner diameters were 0.016 and 0.026 inches. This procedure was conducted at room temperature. Angiography was performed to detect the tumor-supplying vessels and both the microcatheter and microwire were super-selectively catheterized into the tumor-supplying vessels for embolization. The embolization was discontinued after the flow of the contrast agent stopped. Within 5 min of the chemotherapeutic agent delivery, another angiography was performed to determine if the [blushed/tinted] tumor was still visible and if so, the embolization procedure was repeated. One or two vials (maximum two vials) of DC beads were used in all procedures. Gelpart (1 mg; Nippon Kayaku Co. Ltd., Tokyo, Japan) was administered during arterial embolization if a vascular lake was detected in the tumor.

### 2.3. cTACE Procedures and Timing of Additional cTACE

Angiography was performed to detect the tumor-supplying vessels and percutaneous femoral arterial puncture was performed using the Seldinger technique under topical anesthesia [23]. Subsequently, the microcatheter (1.7Fr; Breakthrough^TM^, Boston Scientific, Marlborough, MA, USA) and microwire (0.016 inch; ASAHI Meister, ASAHI INTECC Co., ltd., Seto, Japan) were super-selectively catheterized into the tumor-supplying vessels for the delivery of the chemotherapeutic reagent, namely a solution of cisplatin (Nichi-iko Pharma Co. Ltd., Toyama, Japan), miriplatin (Dainippon Sumitomo Pharma Co., Ltd., Osaka, Japan) or epirubicin and lipiodol (Guerbet Japan Co. Ltd., Tokyo, Japan). Under radiographic guidance, the infusion was discontinued when the flow of lipiodol stopped. Another angiography was performed to ensure that lipiodol had been deposited and to confirm adequate infusion. Gelpart (1 mg; Nippon Kayaku Co. Ltd., Tokyo, Japan) was administered after the transarterial infusion of lipiodol with the chemotherapeutic reagents until adequate embolization. Second or third cTACE was performed in 2 months after DEB-TACE.

### 2.4. Treatment Outcome Assessment Criteria

Treatment outcomes were assessed within 1–3 months after the first cycle of DEB-TACE and again after the second or third cycle of cTACE according to imaging results and the Response Evaluation Criteria in Cancer of the Liver version 5.0, as follows. (i) A complete response: the loss of any intratumoral arterial enhancement in all target nodules; (ii) a partial response: at least a 30% decrease in the sum of the diameters of viable (enhancement in the arterial phase) target nodules relative to the baseline sum of the diameters of target nodules; (iii) a stable disease: absence of partial response or progressive disease; (iv) a progressive disease: an increase of at least 20% in the sum of the diameters of viable (enhancing) target nodules relative to the smallest sum of the diameters of viable (enhancing) target nodules recorded since treatment started; (v) an objective response rate: the percentage of patients who achieved a complete response or partial response; and (vi) a disease control rate: the percentage of patients who achieved a complete response, partial response, or stable disease [24].

### 2.5. Liver Function and Safety Assessment

Liver function was assessed using liver function-related laboratory parameters including albumin, total bilirubin, alanine aminotransferase, and aspartate aminotransferase. All adverse events including pain, fever, nausea, and vomiting were recorded using the Common Terminology Criteria for Adverse Events (CTCAE c5.0) [25].

### 2.6. Statistical Analyses

GraphPad Prism version 8.4.2 (GraphPad Software, San Diego, CA, USA) was used for the statistical analyses. Data are presented as count (%), mean ± standard deviation, or median (25–75th). A comparison between the two treatment groups was performed by the chi-square test. The Student’s *t*-test was used to compare numerical data for each group. A value of *p* < 0.05 was considered significant. Univariate analyses for continuous variables were undertaken using the Student’s *t*-test, paired *t*-test, and one-way ANOVA. For the analysis of categorical variables, the Mann–Whitney U test, Fisher’s exact test, chi-squared test, proportional hazard model test, and Gray’s test with log-rank test results were performed. A multivariate analysis was performed using the Cox proportional hazards model and was applied only to variables that were statistically *p* < 0.05 in the univariate analysis. A survival analysis was performed using the Kaplan–Meier method.

## 3. Results

### 3.1. Course of Treatment

**Figure 1:**
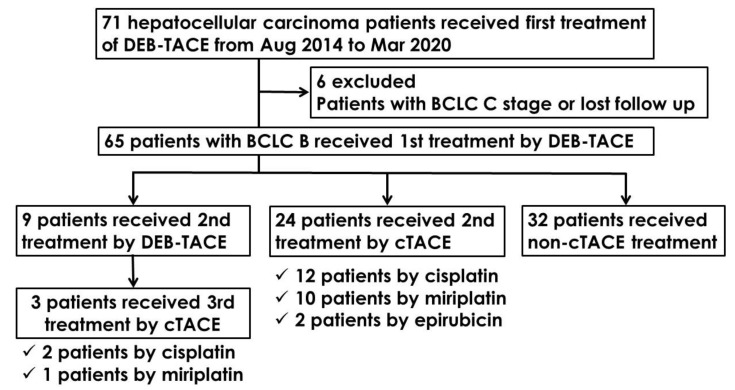
Flow chart of treatment received by the 65 patients enrolled in our study.

As shown in Figure 1, after seventy-one study participants received their first DEB-TACE treatment, six patients were excluded from the study due to having the BCLC C stage of liver cancer or loss to follow-up. The final study population consisted of 65 patients. Of these, nine patients received a second DEB-TACE treatment. Three patients from this group of nine received a third cTACE treatment. Twenty-seven patients received a second or third cTACE treatment: fourteen patients using cisplatin, eleven patients using miriplatin, and two patients using epirubicin. Thirty-eight patients underwent non-cTACE treatment, including six patients with a second DEB-TACE treatment.

### 3.2. Patient Characteristics

The baseline characteristics of the patients with HCC are summarized in Table 1. There were no significant differences between the cTACE and non-cTACE groups with respect to gender, age, etiology (HBV or HCV/NBNC), Child-Pugh score (5 or 6/7 or 8), clinical stage (II or III/IVA or IVB), tumor number (<4/4≤), maximum tumor size, MC (SN or SNE/CMN), up-to-7 criteria (IN/OUT), AFP, AFP-L3, or DCP. The cTACE group consisted of eighteen male and nine female patients with a median age of seventy-five (range: 54–89) years. The non-cTACE group consisted of thirty-one male and seven female patients with a median age of seventy-eight (range: 54–90) years (Table 1). The number of patients with tumors <4 cm and ≥4 cm in size were twenty (74.1%) and seven (25.9%) in the cTACE group, respectively, and thirty-four (89.5%) and four (10.5%) in the non-cTACE group, respectively (Table 1). The median tumor size was 59 mm (range: 30–131) in the cTACE group and 69 mm (range: 12–200) in the non-cTACE group. In the cTACE group, twenty-three patients (85.2%) had single nodular-type lesions or those with extranodular growth and four patients (14.8%) had confluent multinodular-type lesions. In the non-cTACE group, thirty-one patients (81.6%) had single nodular-type lesions with or without extranodular growth and seven patients (18.4%) had confluent multinodular-type lesions (Table 1).

### 3.3. Treatment Responses

**Figure 2:**
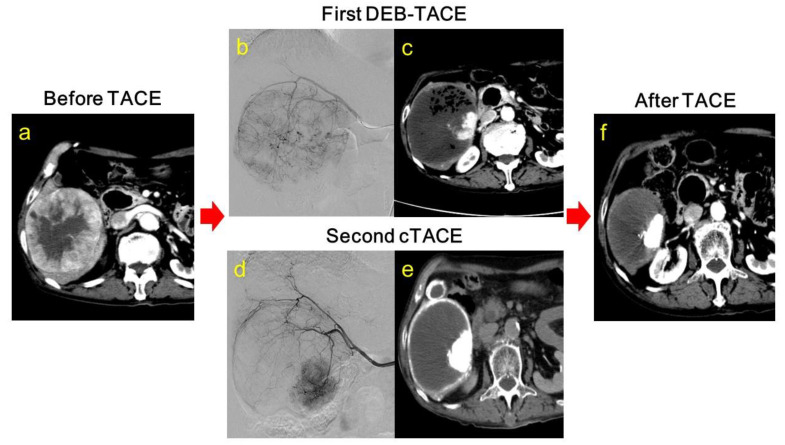
Radiographic images showing progress of treatment in 54-year-old female study participant with hepatocellular carcinoma: (**a**) dynamic computer tomography image from early treatment phase before transcatheter arterial chemoembolization (TACE) procedures; (**b**) angiographic image taken during the first drug-eluting beads-transcatheter chemoembolization (DEB-TACE) procedure; (**c**) dynamic computer tomography image from the early treatment phase a month after the first DEB-TACE procedure; (**d**) angiographic image taken during the second conventional TACE (cTACE) procedure; (**e**) regular computer tomography image taken immediately after the second cTACE procedure; and (**f**) dynamic computer tomography image taken a month after the second cTACE procedure.

In the cTACE group, after the first cycle of DEB-TACE, no patient and 26 patients (96.3%) achieved a complete response and partial response (Figure 2a–c), respectively, resulting in an overall response rate of 96.3% and a disease control rate of 100% (Table 2). In the non-cTACE group, the numbers of patients who achieved a complete response and partial response were 1 (2.6%) and 32 (84.2%), respectively, resulting in an overall response rate of 86.8% and a disease control rate of 86.8% (Table 2). No statistically significant difference was detected between the cTACE and non-cTACE groups after the first DEB-TACE.

We also examined the effect of additional cTACE on the treatment outcome of the first DEB-TACE or second DEB-TACE. In the non-cTACE group, four patients (10.5%) achieved a complete response and twenty-four patients (63.2%) achieved a partial response after the first DEB-TACE or second DEB-TACE, while fourteen patients (51.9%) and eight patients (29.6%) achieved a complete response (Figure 2d,e,f) and partial response, respectively, in the cTACE group (Table 3). Interestingly, the complete response rate in the cTACE group was significantly higher than that in the non-cTACE group (*p* *=* 0.0002). The overall response rate and disease control rate were not significantly different between the groups (both *p* > 0.05; Table 3).

### 3.4. Factors Contributing to Complete Response to DEB-TACE Followed by cTACE

Our analysis of various factors related to the complete response to DEB-TACE followed by cTACE is shown in Table 4. Based on our analysis, the following factors did not contribute: age, Child-Pugh score, etiology, tumor size, MC, AFP, AFP-L3, and DCP. No difference between cTACE with cisplatin and that with miriplatin/epirubicin contributed to the complete response. Remarkably, tumor number alone (<4/≥4) contributed to the CR rate, in contrast to non-CR [partial response (PR), stable disease (SD), and progressive disease (PD)] rate (Table 4). These results suggest that DEB-TACE followed by cTACE is highly effective for HCC patients with less than four liver tumors.

### 3.5. Comparison of Overall Survival Time between CR and Non-CR (PR + SD + PD) Patients in the cTACE Group

**Figure 3:**
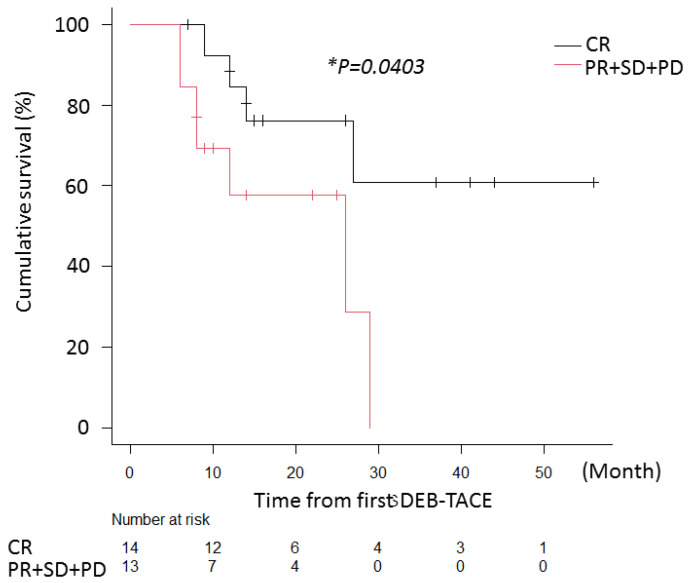
Cumulative rates of overall survival: the Kaplan–Meier method and log-rank test were used to assess the cumulative rates of overall survival. The black line indicates patients within the cTACE group and the red line indicates patients within the non-cTACE group (* *p* < 0.05).

The comparison of the overall survival time between CR and non-CR (PR+SD+PD) patients in the cTACE group were analyzed. The median observation time was 14 ± 13.1 months after the first DEB-TACE. During the follow-up period, twenty-six patients (96.3%) died and one patient (3.7%) was still alive at the end of the observation. Among the patients with CR, thirteen patients (92.9%) died and one patient (7.1%) was still alive; among the cases with non-CR, thirteen patients (100%) died (Figure 3). Furthermore, there was a significant difference in the overall survival (OS) between CR and non-CR patients in the cTACE group (* *p* = 0.0403, Figure 3).

### 3.6. Complications and Adverse Events

One week after the additional cTACE, nine patients (33.3%) experienced general fatigue, nine (33.3%) experienced appetite loss, twelve (44.4%) experienced fever, eight (29.6%) experienced pain, two (7.4%) had ascites, three (11.1%) had anemia, five (18.5%) had thrombocytopenia, four (14.8%) had hyperbilirubinemia, and twenty-seven (100%) had liver dysfunction, while only one (3.7%) patient had severe thrombocytopenia (grade 3) (Table 5). One month after the additional cTACE, two patients (7.4%) had ascites and no other adverse events were observed.

## 4. Discussion

Several clinical trials have demonstrated the efficacy and safety of DEB-TACE. A multicenter phase 2 randomized trial using DEB-TACE with doxorubicin-eluting beads (PRECISION V) showed a marked reduction in liver toxicity and drug-related adverse events as compared to that using cTACE with doxorubicin [16,26,27]. Our study also showed that DEB-TACE was efficient and safe (Table 2). Lammer et al. have also demonstrated that in patients with liver cancer, the tumor response is greater after DEB-TACE than cTACE [26,28]. Golfieri and colleagues conducted a randomized trial of DEB-TACE vs. cTACE in 177 patients with HCC. DEB-TACE and cTACE were found to be equally effective and safe, with the only advantage of DEB-TACE concerning the fact that it induced less postoperative abdominal pain [29]. The rationale behind DEB-TACE is that drug-loaded embolic microspheres help expose the target tumor to the antineoplastic drug for an extended period of time, while reducing the systemic circulation of the drug, resulting in lower toxicity for the patient [16]. Existing data therefore support our finding that DEB-TACE is useful for treating the intermediate stage of HCC. Nevertheless, there is no clinical evidence of DEB-TACE’s possible advantage over cTACE. A randomized control study on this topic failed to show that DEB-TACE was superior to cTACE, with the two-year survival rates being 56.8% and 55.4%, respectively [29]. Conversely, the CR rate of DEB-TACE was higher than that of cTACE, but the PR rate also remained high [30]. In addition, OS with DEB-TACE was not prolonged compared to OS with cTACE [31]. In our study, when we performed cTACE 2 months after DEB-TACE, the CR rate was clearly increased. Therefore, we hypothesized that the embolization sites of DEB-TACE and cTACE might be different, and this combination might be effective for the treatment of unresectable hepatocellular carcinoma. In fact, among several cases of liver tumor of the single or multiple nodular type, viable parts of the tumor are often detected after DEB-TACE (Figure 2b). The remaining viable parts are due to the characteristics of DEB-TACE, regarding the fact that no drug-eluting beads reach the peribiliary vascular plexus [32]. This drainage area around the tumor is the first metastatic route of hepatocellular carcinoma and micrometastasis is detected at a high rate, and treatment similar to that of the cancerous part is desired [32]. In contrast, in cTACE, lipiodol injected from the hepatic artery fills the intratumoral sinus according to hemodyamics and accumulates in the drainage area [19]. We addressed that issue by performing an additional cTACE procedure by passing lipiodol through the vasculature to be accumulated in the tumor’s drainage area (Figure 2f), which in turn might lead to an accumulation of lipiodol in the non-tumorous hepatic parenchyma around the liver tumor [19,33,34,35,36]. Our results demonstrated that DEB-TACE remarkably increased the complete response rate among the intermediate stage HCC patients (Table 3). Extrapolating from that finding, combined therapy with DEB-TACE followed by cTACE might also induce necrosis in the intermediate stage of HCC in a complementary manner.

Our data demonstrated that DEB-TACE followed by cTACE may be promising in the treatment of the intermediate stage of HCC, as it achieves a higher complete response rate and induces major tumor necrosis, while reducing the side effects of chemotherapy (Table 5). Additionally, the OS rate of CR patients was higher than that of non-CR patients in the cTACE group (Figure 3). In our study, we chose DEB-TACE as the first of two combined therapies because it is associated with less liver toxicity and fewer drug-related adverse events than cTACE, even though its therapeutic effect may be insufficient. In fact, Kalayci et al. showed that the area under the curve and peak concentration levels were the same between systemic chemotherapy and cTACE; thus, the value of cTACE is complicated by the side effects of chemotherapy [37]. With DEB-TACE, a lower amount of chemotherapeutic drugs pass through the patient’s circulatory system, even when these drugs are locally injected in very high doses. Our study confirmed that the area under the curve and the peak concentration values produced by DEB-TACE were significantly lower than those produced by cTACE.

A therapeutic limitation of DEB-TACE concerns its possible insufficient embolization of the liver tumor. Drug-eluting beads are loaded into the peribiliary plexus, the main feeder of the bile duct wall. This could damage the area by decreasing arterial blood flow or by chemical insult to the vessel walls caused by highly concentrated antitumor drugs [32]. However, no drug-eluting beads accumulate in the tumor’s drainage area, including non-tumorous hepatic parenchyma around the HCC. Therefore, cTACE may be critical for the treatment of the intermediate stage of HCC considering lipiodol in the non-tumorous liver adjacent to the tumor may correspond to the drainage area.

The present study has some limitations. The number of patients enrolled was limited and the follow-up duration was short. However, our findings concerning DEB-TACE followed by cTACE, which achieved a high CR rate and prolonged OS for CR patients as compared to non-CR patients in the cTACE group, are noteworthy. Therefore, our new combination therapy might be quite valuable for the treatment of the intermediate stage of HCC. Further research for the long-term outcomes with many participants might enable us to obtain the prolonged survival time of patients with intermediate stage HCC.

## 5. Conclusions

DEB-TACE has recently been performed for intermediate stage HCC. However, the residual viable lesion located in the drainage area is often observed after DEB-TACE. cTACE involves accumulating lipiodol-containing anticancer drugs in the drainage area, which is the first site of HCC recurrence. Therefore, in our present study, DEB-TACE DEB-TACE followed by cTACE was able to reduce the side effects of chemotherapy while increasing the CR rate, which is only affected by the total number of tumors, and prolonge the OS of HCC patients who reached CR. This combination therapy appears to be promising for the treatment of the intermediate stage of HCC.

## Figures and Tables

**Table 1:** Baseline characteristics of the patients examined in the study.

Parameters	*n* = 27, cTACE(+)	*n* = 38, cTACE(−)	*p-*Values
Age, median (range)	75 (54–89)	78 (54–90)	0.3286
Sex (male/female)	18/9	31/7	0.1375
Etiology (HBV or HCV/NBNC)	11/16	23/15	0.1155
Child-Pugh score (5, 6/7, or 8)	21/6	23/15	0.1428
cStage (II/III)	8/19	19/19	0.1288
Tumor number (<4/4≤)	20/7	34/4	0.1027
Maximum tumor size (mm), median (range)	59 (30–131)	69 (12–200)	0.7136
MC (SN or SNE/CMN)	23/4	31/7	0.7024
up-to-7 criteria (IN/OUT)	14/13	26/12	0.1760
AFP (ng/mL), median (range)	9192 (4–149,280)	19,672 (2–250,434)	0.9038
AFP-L3 (%), median (range)	19 (0.1–82)	32 (0.5–86)	0.6389
DCP (mAU/mL), median (range)	9594 (30–68,034)	39,178 (8–816,823)	0.3717

Abbreviations: HBV, hepatitis B virus; HCV, hepatitis C virus; cStage, clinical tumor stage; MC, macroscopic classification; SN, single nodular type; SNE, single nodular type with extranodular growth; CMN, confluent multinodular type; AFP, α-fetoprotein; and DCP, des-γcarboxy prothrombin.

**Table 2:** Treatment response to first DEB-TACE.

Parameters	cTACE(+), *n* = 27	cTACE(−), *n* = 38	*p-*Values
Complete response (CR)	0 (0)	1 (2.6)	0.3959
Partial response (PR)	26 (96.3)	32 (84.2)	0.1214
Stable disease (SD)	1 (0)	0 (0)	0.2319
Progressive disease (PD)	0 (0)	5 (13.2)	0.0500
Overall response rate (ORR)	26 (96.3)	33 (86.8)	0.1944
Disease control rate (DCR)	27 (100)	33 (86.8)	0.0500

Data are presented as count *n* (%).

**Table 3:** Treatment response to additional cTACE.

Parameters	cTACE(+), *n* = 27	cTACE(−), *n* = 38	*p-*Values
Complete response (CR)	14 (51.9)	4 (10.5)	* 0.0002
Partial response (PR)	8 (29.6)	24 (63.2)	* 0.0077
Stable disease (SD)	1 (3.7)	2 (5.3)	0.7678
Progressive disease (PD)	4 (14.8)	8 (21.1)	0.5230
Overall response rate (ORR)	22 (81.5)	28 (73.7)	0.4622
Disease control rate (DCR)	23 (85.2)	30 (78.9)	0.5230

Data are presented as count *n* (%). cTACE(−) excluded second DEB-TACE (* *p* < 0.05).

**Table 4:** Factors contributing to CR by DEB-TACE following cTACE (CR vs. non-CR).

	Uni-Variate Analysis				Multi-Variate Analysis			
Parameters	OR	95% CI		*p*-Values	OR	95% CI		*p*-Values
Age (<80/80≤)	0.00772	−0.01404	0.02948	0.4717	0.01196	−0.01671	0.04064	0.3896
Child-Pugh (A/B)	0.02381	−0.47115	0.51876	0.92187	0.10314	−0.59448	0.80076	0.758
Etiology (HBVorHCV/NBNC)	0.04546	−0.373	0.46391	0.82479	0.05984	−0.45035	0.57003	0.8068
Tumor number (<4/4≤)	0.50714	0.08651	0.92777	* 0.02009	0.69165	0.08865	1.29464	* 0.02716
Tumor size (<75/75≤)	0.12143	−0.34555	0.58841	0.59701	0.28535	−0.31613	0.88683	0.32953
MC (SN+SNE/CMN)	−0.27174	−0.84018	0.2967	0.33428	0.06001	−0.72434	0.84435	0.8732
AFP (ng/mL) (<9/9≤)	0.1	−0.4282	0.6282	0.69992	−0.81159	−3.33532	1.71213	0.50516
AFP-L3 (%) (<11/11≤)	0.10989	−0.29952	0.51930	0.58531	0.08614	−0.37755	0.54983	0.6989
DCP (mAU/mL) (<3700/3700≤)	0.18681	−0.21784	0.59147	0.35081	0.23685	−0.32434	0.79804	0.3842
cTACE (Cisplatin or Miriplatin/Epirubicin)	−0.03846	−0.45007	0.37314	0.84894	0.08178	−0.41276	0.57632	0.7305

Odds-ratios for continuous variables were calculated for one unit. (* *p* < 0.05).

**Table 5:** Safety profile following the additional cTACE (*n* = 27).

CTCAE v5.0	All Grades *n* (%)	Grade3 *n* (%)
General fatigue	9 (33.3)	0
Appetite loss	9 (33.3)	0
Fever	12 (44.4)	0
Pain	8 (29.6)	0
Ascites	2 (7.4)	0
Anemia	3 (11.1)	0
Thrombocytopenia	5 (18.5)	1 (3.7)
Hyperbilirubinemia	4 (14.8)	0
Liver Dysfunction	27(100)	0

Data are presented as count *n* (%). Abbreviation: CTCAE v5.0, Common Terminology Criteria for Adverse Events version 5.0.

## Data Availability

The data used in the present study are available from the corresponding author upon reasonable request.
